# Myocardial perfusion mapping with intra‐arterial spin labeling: Optimization of the labeling efficiency

**DOI:** 10.1002/mrm.30589

**Published:** 2025-06-04

**Authors:** Felix Spreter, Johannes Fischer, Ali Caglar Özen, Alexander Maier, Michael Bock, Simon Reiss

**Affiliations:** ^1^ Division of Medical Physics, Department of Diagnostic and Interventional Radiology, University Medical Center Freiburg, Faculty of Medicine University of Freiburg Freiburg Germany; ^2^ Department of Cardiology and Angiology, University Heart Center Freiburg‐Bad Krozingen, Faculty of Medicine University of Freiburg Freiburg Germany

**Keywords:** ASL, cardiac, interventional, MRI

## Abstract

**Purpose:**

Intra‐arterial spin labeling (iASL) applies a labeling pulse from a transmit coil at the tip of the catheter for regional myocardial perfusion measurements during MR‐guided catheterizations. This study investigates the labeling efficiency of catheter‐mounted coils under varying conditions to enable robust iASL measurements.

**Methods:**

The labeling efficiency was assessed by analytical calculations, numerical simulations, and in vitro measurements at 3 T. Analytical solutions were provided for various simplified $B_{1}^{+}$ field distributions, and a numerical simulation was used to calculate $B_{1}^{+}$ for different catheter coil designs using the Biot‐Savart Law. Using these $B_{1}^{+}$ fields, the RF excitation of the blood magnetization flowing around the catheter was calculated using the Bloch equation. Finally, in vitro measurements were performed in a flow phantom using loop and solenoid catheter coils.

**Results:**

$B_{1}^{+}$ fields produced by the catheter coils result in a saturation of the mean magnetization when the applied pulse exceeds a threshold power. This threshold power varies between 1 and 25 mW for different coil designs, flow velocities, and geometric parameters.

**Conclusion:**

iASL creates a saturation that is robust against variations in flow and coil positioning. The simulations could determine the threshold pulse power for robust iASL excitation, which needs to be adapted to applications in different vessels. Thus, iASL may provide an efficient alternative to perfusion measurements with exogenous contrast agents during MR‐guided interventions.

## 1 INTRODUCTION

Cardiac catheterizations for the diagnostics and treatment of coronary artery disease are commonly performed under X‐ray fluoroscopy guidance. Recent advances in devices and imaging sequences have enabled MRI‐guided coronary catheterizations in preclinical studies.^1^, ^2^, ^3^, ^4^ The use of MRI is advantageous because MRI lacks ionizing radiation and offers superior soft tissue contrast and 3D imaging. It thus enables comprehensive imaging of cardiac function and perfusion, which could help monitor the treatment success during the MR‐guided intervention.

Active catheter visualization and tracking are key techniques to make MR‐guided catheterizations feasible, particularly in small vessels and in close proximity to the heart. Markers are attached to the catheter, which are visible under real‐time imaging. Active markers consist of a small RF coil, which is connected to the MRI system as a receive coil so that the tissue or blood surrounding the coil is visible as hyperintensity.^1^, ^2^, ^5^, ^6^, ^7^, ^8^ Besides visualization, these coils have also been used for device tracking and blood‐flow velocity measurements.^9^

RF coils at the tip of a catheter can also be operated as transmit (Tx) coils to perform intra‐arterial spin labeling (iASL). iASL is an adaptation of arterial spin labeling (ASL), for which the labeling pulse is applied from inside the artery: An RF pulse is applied at the Larmor frequency to selectively label the blood around the coil. In the tissue downstream of the coil, the labeled blood reduces the MRI signal; and after subtraction of an image without labeling (reference image), a semi‐quantitative perfusion map can be generated. Initially, the feasibility of iASL was demonstrated in an in vitro experiment and in vivo in two animals.^10^ However, only one type of catheter coil was used at one specific labeling power. The complex labeling process via the inhomogeneous B_1_ field of the catheter coils has yet to be studied, and is needed to further optimize the iASL technique.

When performed in coronary arteries, iASL allows for myocardial perfusion measurements without the use of an exogenous contrast agent (CA). Thus, measurements can be repeated arbitrarily during a coronary intervention and enable perfusion measurements in patients with contraindications for gadolinium‐based CAs.^11^ So far, ASL is well established for brain imaging but faces challenges in cardiac applications. The most widely used implementation of cardiac ASL is flow‐sensitive inversion recovery,^12^, ^13^, ^14^, ^15^, ^16^ for which global and slice‐selective labeling pulses create a perfusion measurement in a central short axis slice. Compared to flow‐sensitive inversion recovery, iASL allows for volumetric imaging and displays the perfusion through one specific coronary artery of interest. Conceptually closer to iASL is velocity‐selective myocardial ASL^17^, ^18^; blood flowing with a velocity larger than a cutoff velocity is labeled by saturation of the magnetization and proximal aorta tagging,^19^ where blood is directly labeled inside the aortic root.

For ASL measurements, it is important to achieve sufficient and robust labeling under varying conditions, especially when perfusion is quantified.^20^ Robust labeling is challenging to achieve because blood flow and coil position can change, and the Tx $B_{1}^{+}$ field of the catheter coil is inherently inhomogeneous.

This study investigates the labeling efficiency of iASL to determine the signal difference that can be achieved and to allow for optimization of the coil design and labeling pulse. The labeling process is analytically calculated for a simplified flow pattern and field distribution, and a numerical simulation is set up to compare different coil designs and blood flow conditions. Various coils are built and tested in vitro.

## 2 METHODS

### 2.1 Simulation of labeling

The labeling efficiency was calculated analytically for simplified models of rotationally symmetric labeling fields that decrease with the distance to the coil center (see Figure S1). Because labeling with realistic $B_{1}^{+}$ field distributions can only be calculated numerically, a simulation framework was set up, which can be accessed on GitHub (see Data Availability Statement). First, the geometry of the catheter coil was defined to determine the $B_{1}^{+}$ field using MatLab (R2024a, MathWorks, Natick, MA). The coil was assumed to be constructed from thin wire (Ø 0.15 mm) wound on the surface of a cylinder (Ø 2 mm). The RF current amplitude I during labeling is approximated to be constant along the wire because the total wire length is small compared to the wavelength of the RF field (⌊ = 26 cm). The wire was divided into N small segments, and for each segment the position ${\vec{r}}_{i}$ and direction ${\vec{c}}_{i}$ of the current is used to calculate the B field using the law of Biot‐Savart. A simulation volume was defined over a length of 2 cm along the cylinder axis, 1 × 1 cm^2^ in the cross‐section of the tube, and with the coil at the center. The volume was divided into voxels of 0.1 mm, and the B field for each voxel (*k,l,m*) is calculated by:

(1) $${\vec{B}}_{k,l,m}=\sum_{i=1}^{N}I\;{\vec{c}}_{i}\times \frac{{\vec{r}}_{k,l,m}^{\prime }-{\vec{r}}_{i}}{{\left|{\vec{r}}_{k,l,m}^{\prime }-{\vec{r}}_{i}\right|}^{3}}.$$

Only the components of ${\vec{B}}_{k,l,m}$ that are orthogonal to $B_{0}$ contribute to the excitation of the magnetization—here, these are the x‐ and y‐ components. Furthermore, the angle θ between the catheter and $B_{0}$ is introduced by taking the dot product with the vector (cos(θ), sin(θ), 0). Because only one circular polarized component is considered, a factor of ½ is introduced, yielding

(2) $${\vec{B}}_{1_{k,l,m}}^{+}=\frac{1}{2}\left(\begin{array}{l} \cos\left(\theta \right)\\ \sin\left(\theta \right)\\ 0 \end{array}\right)\cdot {\vec{B}}_{k,l,m}.$$

Then, the flow of the blood magnetization through the volume is defined using streamlines, assuming straight and concentric artery and catheter. The coil is positioned in the entry region of the coronary artery, approximating constant velocity over the vessel cross‐section.^21^ The mean flow velocity in coronary arteries varies over the cardiac cycle,^22^ but these variations are slow compared to the time the blood takes to traverse the labeling field (for v=10 cm/s, l=1 cm, passage duration = 100 ms); therefore, a constant velocity is assumed for individual simulations.

For each voxel, the rotation caused by $B_{1}^{+}$ is calculated from the time ∆t=∆x/v the magnetization takes to traverse the voxel. The rotation is defined by the flip angle $\alpha =\gamma \left|{\vec{B}}_{1}^{+}\right|\cdot ∆t$ and the rotation axis $\vec{n}={\vec{B}}_{1}^{+}/|{\vec{B}}_{1}^{+}|$. This allows for expression of the rotation by the Rodrigues rotation matrix,^23^

(3) $$\begin{array}{cc} & R\left(\alpha ,\vec{n}\right)=\\ & \;\left(\begin{array}{ccc} \cos\alpha +n_{1}^{2}\left(1-\cos\alpha \right) & n_{1}n_{2}\left(1-\cos\alpha \right)-n_{3}\sin\alpha & n_{1}n_{3}\left(1-\cos\alpha \right)+n_{2}\sin\alpha \\ n_{1}n_{2}\left(1-\cos\alpha \right)+n_{3}\sin\alpha & \cos\alpha +n_{2}^{2}\left(1-\cos\alpha \right) & n_{2}n_{3}\left(1-\cos\alpha \right)-n_{1}\sin\alpha \\ n_{1}n_{3}\left(1-\cos\alpha \right)-n_{2}\sin\alpha & n_{2}n_{3}\left(1-\cos\alpha \right)+n_{1}\sin\alpha & \cos\alpha +n_{3}^{2}\left(1-\cos\alpha \right) \end{array}\right). \end{array}$$

The final magnetization along one streamline is determined by consecutively applying the rotations to ${\vec{M}}_{0}=\left(0,0,1\right)$. The magnetization distribution in a vessel cross‐section is plotted in a magnetization map.

The inhomogeneous ${\vec{B}}_{1}^{+}$‐field of the catheter coil is expected to also create an inhomogeneous distribution of the magnetization after labeling. In addition, the mixing of the magnetization of the spins occurs while flowing to the imaging plane. Thus, the mean longitudinal magnetization $\bar{M_{z}}$ is calculated to evaluate the labeling efficiency. The largest signal difference in ASL is created by an inversion ($\bar{M_{z}}=-1$), and the labeling efficiency E is defined relative to this as:

(4) $$E=\frac{1-\bar{M_{z}}}{2}.$$

Considering the inhomogeneous magnetization in iASL, a saturation ($\bar{M_{z}}=0$); thus, E=0.5 is considered optimal. The free parameters in a single simulation are the coil geometry, the angle θ between the catheter axis and $B_{0}$, the flow velocity v, the position of the catheter relative to the vessel, and the current I. The current is related to the labeling pulse power $P=I^{2}R$, assuming R=50Ω.

**FIGURE 1:**
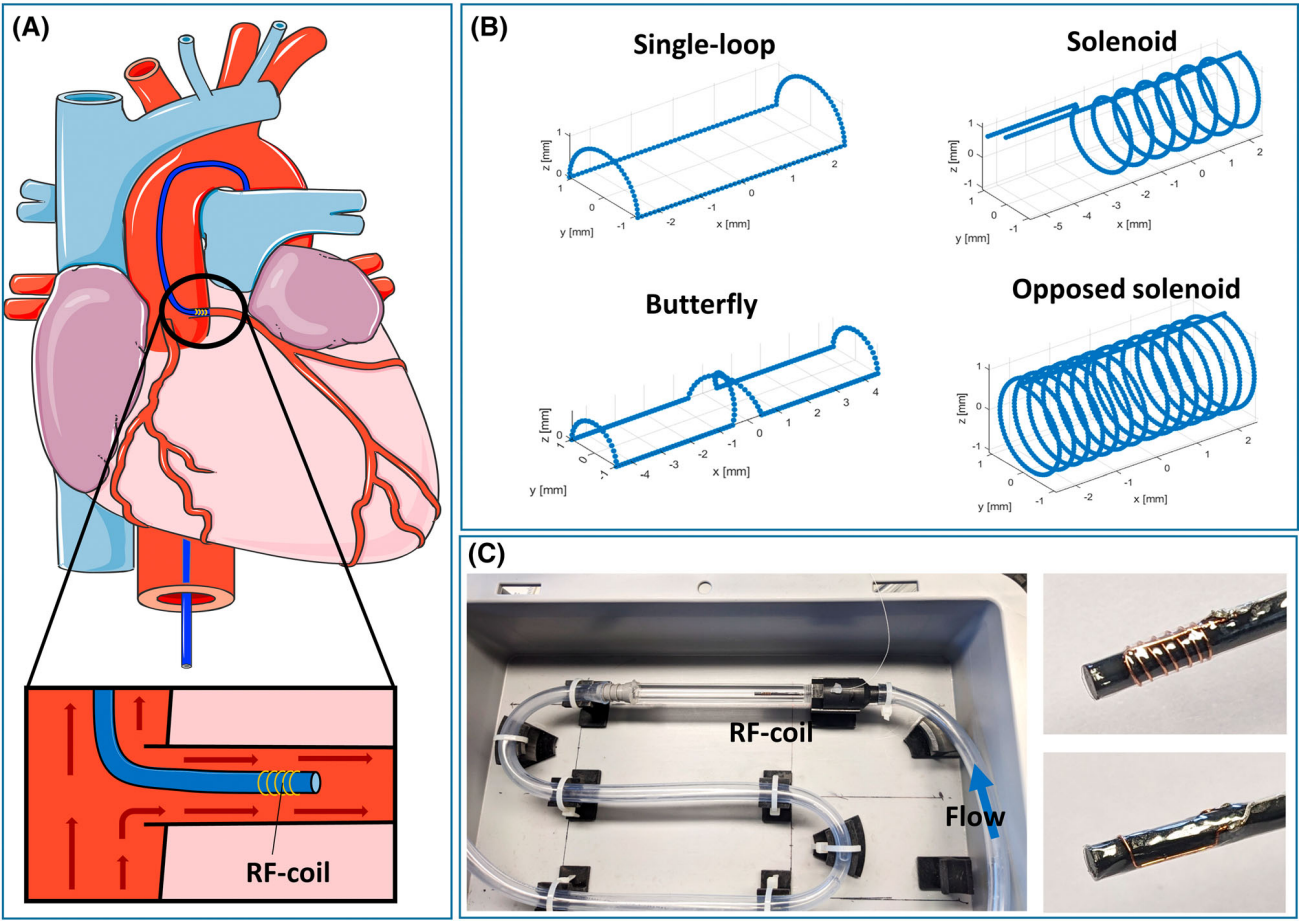
(A) Schematic of the setup for an iASL measurement. An active catheter with an RF coil at the tip is inserted in the target vessel (left coronary artery), and the blood flowing around the catheter is labeled using the coil as a transmit coil. (B) Schematics of four different simulated coil geometries that are attached to a cylindrical catheter (not shown). (C) Flow phantom with water flowing around the coils on the right. The transverse magnetization is measured in an imaging plane 2.7 cm downstream of the coil. iASL, intra‐arterial spin labeling.

First, the simulation was carried out for four coil geometries that could fit on the tip of a 6F catheter: a single‐loop coil (saddle structure, length: 5 mm), a solenoid coil (windings: 7, length: 5 mm), a butterfly coil (saddle structure, length 10 mm), and an opposed solenoid coil (windings: 7 in both directions, length: 5 mm) (Figure 1). For each coil type, pulse powers between 0.008 mW and 80 mW were simulated. The angle θ was set to 70°, corresponding to the approximate orientation between the left coronary artery (LCA) and $B_{0}$ for a patient positioned headfirst supine in the MRI. The flow velocity in the LCA varies over the cardiac cycle and is highest during diastole, where it reaches values of about 30 cm/s. The peak velocities may be increased during hyperaemia.^22^, ^24^ For the coil comparison, the simulated velocity was set to 10 cm/s.

The influence of θ, v, and coil position was investigated for the single‐loop and the solenoid coil by simulating every combination with θ between 0° and 90° in steps of 10°, v between 5 cm/s and 20 cm/s in steps of 5 cm/s, and nine different coil positions (centered, and with an offset of 45% and 90% of the vessel radius in all directions).

### 2.2 In vitro measurements

Experiments were performed with two catheter coils using a flow phantom at a 3 T MRI system (Magnetom PRISMA Fit; Siemens Healthineers, Erlangen, Germany). A coil holder was 3D printed (Prusa SL1; Prusa Research, Prague, Czech Republic), for which a rod (Ø 2 mm) representing the catheter is fixed inside a tube (Ø 6 mm) representing the vessel.

The coil is wound onto the rod using enameled copper wire (Ø 0.15 mm), soldered to a micro‐coaxial cable (Alpha Wire 9446 WH033, Elizabeth, NJ, OD: 50 μm), and fixed with glue (cyanoacrylate) and a thin shrink tube (Nordson Medical, Westlake, OH, thickness: 6 μm), resulting in a final outer diameter of 2.4 mm. The cable is connected to a tuning and matching network, which contains two variable capacitors for matching the impedance to 50 Ω and one variable capacitor for tuning of the coil. The coil is pre‐tuned by adjusting the length of the coaxial cable and then fine‐tuned with the tuning capacitor. The reflection S_11_ at 123.2 MHz and the *Q*‐value were determined with a vector network analyzer (Rhode & Schwarz ZVB 4, Munich, Germany). For all coils, a reflection coefficient *S*_11_ < −25 dB and an unloaded *Q*‐value >20 were achieved.

The tuning and matching network was connected to a coaxial cable led outside the magnet room via a filter plate and connected via an RF switch (Mini‐Circuits ZASWA‐2‐50DRA+, Brooklyn, NY) to an RF generator (Keysight N5171B, Keysight Technologies, Santa Rosa, CA). During the labeling experiment, the RF generator sends a constant signal, and the RF switch is controlled by a trigger signal, given as a time to live signal from the scanner and implemented as a real‐time event in the measurement sequence.

The $B_{1}^{+}$ field was mapped by acquiring multiple 3D images of the coil vicinity with labeling pre‐pulses of varying duration and equal amplitude. In each voxel, the intensity was plotted against the labeling pulse duration, and the minimal duration to achieve a 50% signal reduction was determined. This corresponds to a flip angle of 60° and thus allows for calculation of $B_{1}^{+}$ in this voxel. For these measurements, the coils were placed parallel to $B_{0}$ in a water basin with gadolinium CA ($T_{1}$ = 852 ms). The labeling pulse was added to a 3D FLASH sequence with center‐out acquisition. A total of 15 image datasets were acquired for each coil with pulse durations between 0 and 15 ms, a pulse amplitude of 20 mW, and the following acquisition parameters: acquisition time: 351 s, TR: 4.9 ms, TE: 2.5 ms, flip angle: $15^{{}^{\circ}}$, matrix: 64 × 64, FOV: 37 × 76 mm^2^, slice thickness: 0.58 mm, bandwidth: 580 Hz/px.

For the labeling measurements, a flow phantom was constructed creating gravitationally driven flow inside a tube (ID: 6 mm). The mean flow velocity was adjusted to 10 cm/s, which was verified with a phase contrast flow measurement. Then, the labeling pulse frequency was adjusted to the local resonance frequency at the coil position because the long labeling pulse has a narrow spectrum compared to the fluctuations induced by $B_{0}$ variations.

The imaging plane was positioned orthogonal to the tube and 2.7 cm downstream of the tip of the coil. For each set of parameters, a measurement was conducted for pulse powers between 0.008 and 80 mW. The pulse was applied for 2 s, and immediately after that an image was acquired with a FLASH sequence with the following measurement parameters: 300 ms, TR: 5.21 ms, TE: 2.7 ms, flip angle: $8^{{}^{\circ}}$, matrix: 160 × 120, FOV: 76 × 57 mm^2^, slice thickness: 6.5 mm, bandwidth: 403 Hz/px.

Measurements were conducted for the single‐loop (length: 5 mm) and the solenoid coil (windings: 7, length: 5 mm) for the same parameters as in the simulation at a fixed angle $\theta =70^{{}^{\circ}}$, flow velocity v=10 cm/s, and a centered catheter position in the tube. For all images, the mean signal inside the cross‐section of the tube is calculated and normalized to the mean signal measured when no pulse is applied in order to determine the labeling behavior.

## 3 RESULTS

**FIGURE 2:**
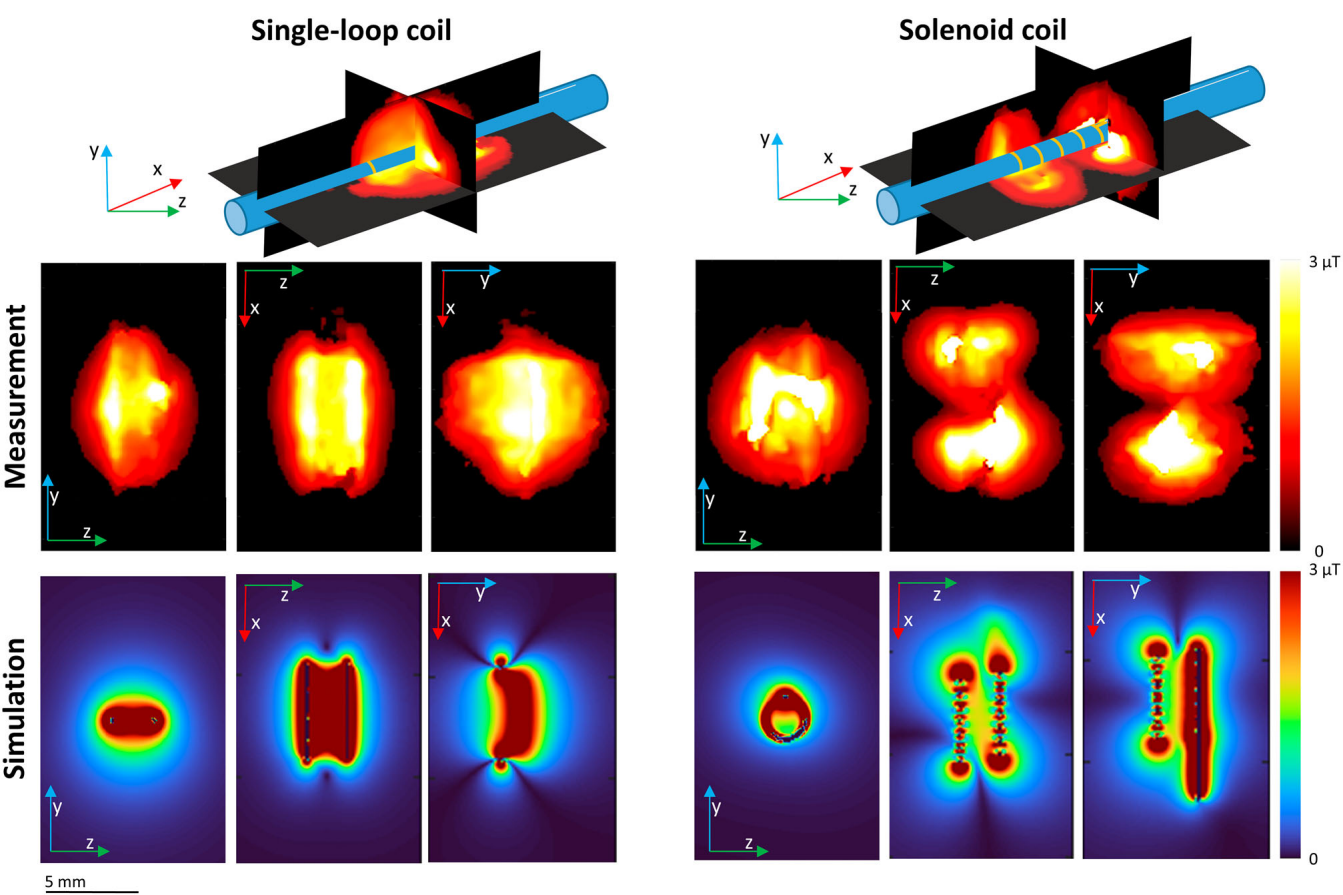
Comparison of measured and simulated $B_{1}^{+}$ field with the catheter orientated parallel to $B_{0}$. (Top) Schematic of the two coils with cross‐sections of the 3D measurement. (Middle) Maximum intensity projections of measured $B_{1}^{+}$. (Bottom) Maximum intensity projections of the simulation $B_{1}^{+}$.

The measured and simulated $B_{1}^{+}$ field for a solenoid and a single‐loop coil with a 20 mW pulse are shown in Figure 2. The mean field in the coil vicinity (Ø 6 mm, length: 10 mm) is 1.0 μT in the measurement and 1.7 μT in the simulation for the solenoid coil. For the single‐loop coil, it is 1.0 μT in the measurement and 1.3 μT in the simulation.

**FIGURE 3:**
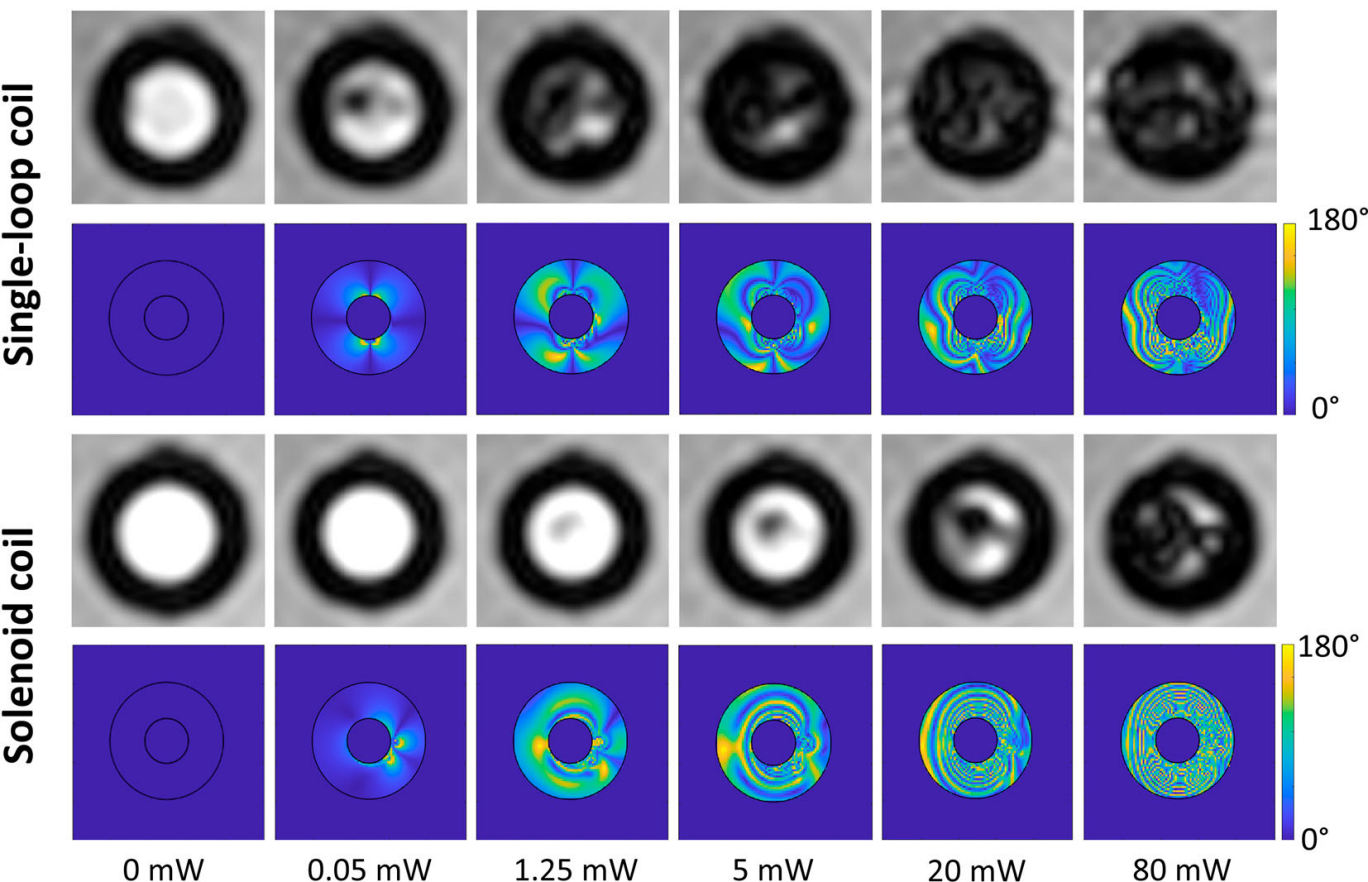
Comparison of the measured signal in a cross‐section of the phantom tube 2.7 cm downstream of the tip of the coil and simulated flip‐angle map for the single‐loop and the solenoid coil at different labeling pulse powers for v=10cm/s and $\theta =70^{{}^{\circ}}$.

Figure 3 shows the simulated effective flip angle map and the measured signal after labeling in a cross‐section of the vessel. For both the single‐loop and the solenoid coil, an inhomogeneous distribution is observed. Even at very low pulse powers of 0.05 mW, the simulation shows that effective flip angles up to 180° are created close to the coil. For higher pulse powers, concentric patterns appear with flip angles between 0° and 180°—these patterns decrease for even larger pulse powers while a homogeneous flip angle distribution is reached. In the measurements, signal voids appear near the center from 0.05 mW for the single‐loop and from 1.25 mW for the solenoid coil. For larger powers, the signal void extends over the whole tube, with some areas remaining bright, especially for the solenoid coil.

**FIGURE 4:**
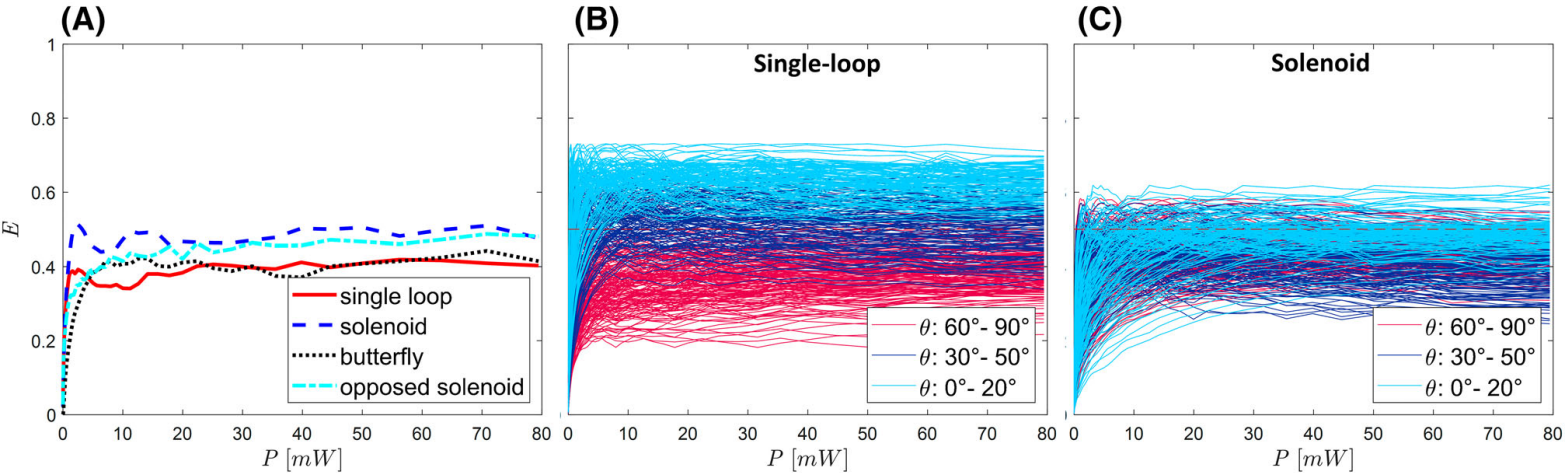
(A) Simulated labeling efficiency *E* as a function of the applied pulse power *P*. For the four different coil geometries in Figure 2 for $\theta =70^{{}^{\circ}}$, v=10 cm/s, and the coil centered in the vessel, with a simulation resolution of 0.1 mm. (B, C) For different coil positions, orientations, and flow velocities. θ was varied between 0° and 90° in 10° steps; v was varied between 5 and 20 cm/s in 5 cm/s steps; and the coil position relative to the vessel was varied between nine different positions. All possible combinations were simulated for the single‐loop and the solenoid coil. Colors indicate the different regimes of the parameter θ in the simulation.

Figure 4A shows the numerical labeling efficiencies comparing the four different coil geometries (single‐loop, solenoid, butterfly, opposed solenoid) for $\theta =70^{{}^{\circ}}$, v=10 cm/s, and a centered coil position. In all geometries, a similar threshold behavior is seen, for which the labeling efficiency increases linearly with increasing power within the first 1.5 mW and then remains approximately constant for higher pulse powers. In Figure 4B,C, the results of *E* for the single‐loop and the solenoid coil are shown for various combinations of θ, v, and positions. For the single‐loop coil, the flat section for larger pulse amplitudes varies between 0.2 and 0.7, which depends strongly on θ. For a pulse of 2 mW, an E above 0.2 is reached for all θ, above 0.38 for θ > 40°, and above 0.55 for θ > 70°. For the solenoid coil, the flat section is between 0.25 and 0.6 and is less dependent on θ. For a pulse of 20 mW, an E above 0.25 is reached for all θ.

**FIGURE 5:**
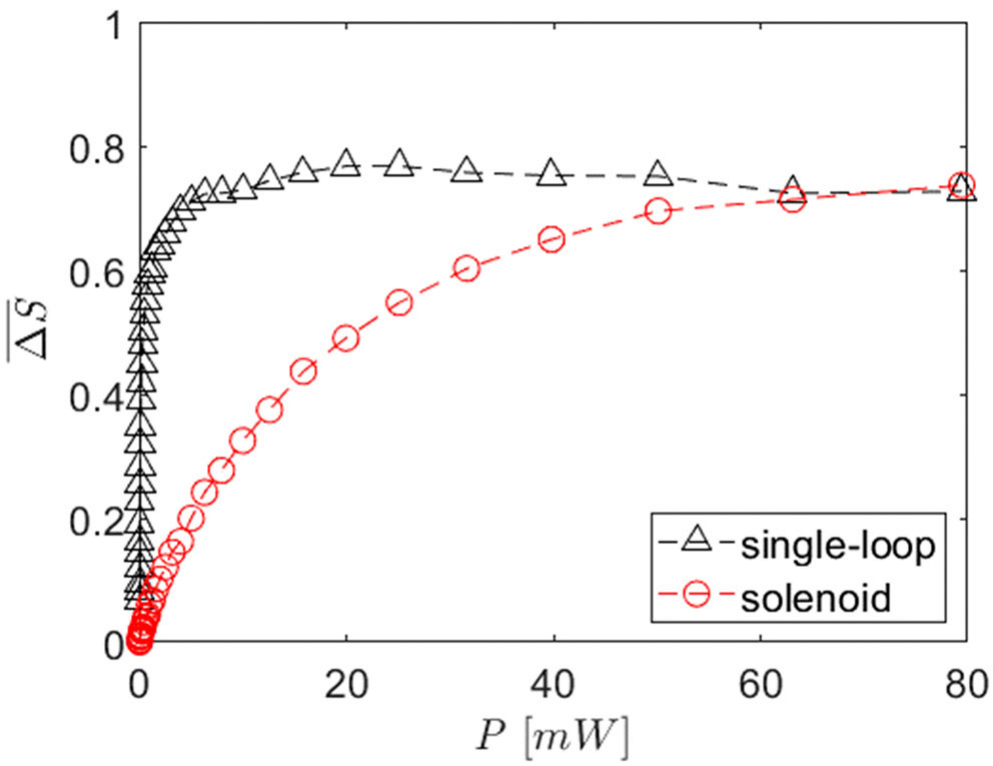
Mean signal difference measured in a cross‐section of the phantom tube 2.7 cm downstream of the coil, as a function of the pulse power, for the single‐loop and the solenoid coil. Measured with θ = 70°, v=10 cm/s, and the coil centered inside the tube.

Figure 5 shows the mean signal intensity measured in a cross‐section after labeling as a function of the labeling pulse power for the single‐loop and the solenoid coil for the same θ, v, and coil position as in Figure 4A. The measured signal intensity for both coils decreases and reaches 0.3 at 5 mW for the single‐loop and 45 mW for the solenoid.

## 4 DISCUSSION

This work investigates the labeling efficiency of catheter‐mounted RF Tx coils for iASL perfusion measurements of the myocardium. Analytical calculations show that the labeling with inhomogeneous fields results in saturations in the limit of large field amplitudes independent of the exact field distribution, in contrast to an oscillatory behavior resulting from a homogenous field.

In general, the $B_{1}^{+}$ field of catheter‐mounted coils is inherently inhomogeneous. Thus, it is expected that the iASL technique achieves a saturation for large $B_{1}^{+}$ amplitudes. This was shown using numerical simulations. The four simulated coil designs show a threshold behavior for the efficiency as a function of the pulse power. For pulses above 5 mW, the efficiency remains approximately constant at *E* = 0.5. A labeling efficiency *E* > 0.5 can only be achieved for specific combinations of pulse power, flow velocity, and coil positioning, which does not appear feasible in a realistic setup.

The minimal pulse power needed for robust labeling is dependent on the coil geometry; the simulations yielded 5 mW for the single‐loop coil and 25 mW for the solenoid coil. Prior knowledge of the setup in which iASL is applied allows for a more precise determination of the expected labeling efficiency; for example, different angles θ result in substantially different efficiencies for the single‐loop coil.

The in vitro measurements confirmed the threshold behavior. A direct comparison of the measurement and the simulation is not possible because the simulated efficiency is calculated from the mean longitudinal magnetization, whereas the measurement is based on the mean signal intensity. However, the measured signal intensity differences of up to 80% clearly indicate that a saturation is feasible using the solenoid and single‐loop coil.

The catheter coils are designed without lumped elements at the tip so that the catheter diameter does not increase substantially. This minimal footprint is vital for interventions but creates unwanted reflections of the applied RF power, reducing the available power of the transmitted $B_{1}^{+}$ field at the tip. This power loss is dependent on the coil geometry and has to be considered when choosing the input RF pulse power. A comparison of the simulated and measured $B_{1}^{+}$‐field amplitudes indicates a greater loss for the solenoid than for the single‐loop coil.

In general, the catheter‐mounted coil and the connecting cable can pose a safety risk due to RF heating. Techniques such as impedance manipulation,^25^ chokes,^26^ or transformers^27^ may be used to reduce RF coupling of the catheter to the RF excitation field. The labeling pulse can cause additional heating: In this study, a pulse power of 25 mW is sufficient to achieve saturation. Assuming that the energy is deposited in a 2 × 2 × 2 cm^3^ volume around the coil, the result would be about 3.1 W/kg, which is below the normal specific absorption rate limit of 4 W/kg.

Limitations to this study include the use of a continuous labeling pulse, which creates a very narrow frequency spectrum for long pulses. The narrow spectrum necessitates a precise adaptation of the applied labeling frequency to the local Larmor frequency at the position of the catheter coil to maximize the efficiency and reduce systematic errors when comparing in vitro measurements to simulations. Pseudo‐continuous labeling schemes could therefore be beneficial to increase the bandwidth and render iASL robust to local frequency shifts. Furthermore, all parameters in this study were chosen for iASL in the LCA. The application of iASL in other vessels and body parts thus requires the adaptation of simulation parameters such as the vessel size, orientation to *B*_0,_ and flow velocity profile. The in vitro measurements and the simulation framework created allow for the adaptation of parameters. However, a limitation of this study is the lack of in vivo measurements. Such measurements will be performed in future animal trials to test the optimized iASL under physiological conditions.

In conclusion, the study provides a comprehensive description of iASL via catheter‐mounted coils. The results reveal that a saturation can be achieved that is robust toward variations in flow and positioning when the pulse power exceeds a threshold on the order of several milliwatts. This saturation is expected to create a significant signal difference between a labeled and unlabeled image in the myocardium and the coronary arteries and could therefore yield a CA‐free perfusion map during an intervention.

## FUNDING INFORMATION

Supported by the German Research Foundation (DFG) under grant RE 4876/1‐1. This study is part of SFB1425 project P15, funded by the Deutsche Forschungsgemeinschaft (DFG, German Research Foundation), 422681845.

## Supporting information


**Figure S1.** Analytically calculated labeling efficiency as function of the mean field strength in the labeling region for three different field distributions (radially symmetric around the vessel center and constant in amplitude and orientation over the length l in z direction). For comparison the simulation results for the same field distributions are shown.

## Data Availability

Simulation source code is available on GitHub: https://github.com/felixspreter/iASL_labeling_efficiency.
