# Supplementary material for: Myocardial perfusion mapping with intra‐arterial spin labeling: Optimization of the labeling efficiency
Source: Magn Reson Med. 2025 Jun 4;94(4):1663–71. doi: 10.1002/mrm.30589 (PMC12309878; doi:10.1002/mrm.30589)
Supplement: Supplementary file 1 — Figure S1. Analytically calculated labeling efficiency as function of the mean field strength in the labeling region for three different field distributions (radially symmetric around the vessel center and constant in amplitude and orientation over the length l in z direction). For comparison the simulation results for the same field distributions are shown. [file MRM-94-1663-s001.docx]

Supplementary Material

Analytical Calculation of Labeling Efficiency:

To calculate the labeling efficiency $E$ analytically, a simplified model is used where a catheter with radius $r_{1}$ is placed centrally in a vessel with radius $r_{2}> r_{1}$. The blood flow is assumed to have a constant special velocity profile, which is an approximation of the flow in the entry region of a coronary artery^20^. For each magnetization $\vec{M}_{0}(x,y)$ in a vessel cross-section that is passing through the transmit field of the coil, the final magnetization after the coil $\vec{M}(x,y)$ is calculated. Therefore, the differential rotations $\boldsymbol{R}\left( \alpha\right)$ caused by the transmit field $\vec{B}_{1}\left( x,y,z \right)$are consecutively applied to the initial magnetization $\vec{M}_{0}(x,y)$ at each position z over the range $l$ that is given by the size of the $\vec{B}_{1}$ field (here: 2-3 times the size of the catheter coil).

| $\vec{M}(x,y;t)=\prod_{z=0}^{l} \boldsymbol{R}\left( {\gamma\vec{B}}_{1}\left( x,y,z \right)t \right)\cdot\vec{M}_{0}(x,y;t=0)$ | (S1) |
| --- | --- |

Here $\gamma$ is the gyromagnetic ratio of the nucleus. This can be calculated for each magnetization passing through the field and the mean longitudinal magnetization of the labeled blood $\overline{M_{z}}$ is then determined by averaging over the vessel cross section and normalizing to the initial magnetization oriented along the z-axis:

| $\overline{M_{z}}(x,y)=\frac{1}{{\pi(r}_{2}^{2}-r_{1}^{2})}\int_{0}^{2\pi} \int_{r_{1}}^{r_{2}} {r \vec{e}}_{z}\cdot\vec{M}(r,\phi;t) d\phi dr$ | (S2) |
| --- | --- |

For arbitrary transmit fields $\vec{B}_{1}\left( x,y,z \right)$ the rotations in Equation (S1) are about different axis, therefore the rotation matrices do not commute and the equation cannot be solved directly. To obtain an approximate analytical solution, the orientation of $\vec{B}_{1}$ is assumed to be constant along the z-axis. This leads to a constant axis for all rotations in Equation (S1) allowing to introduce a total rotation angle $\alpha$. This can be determined by integrating the $\vec{B}_{1}$ amplitude when assuming a constant flow velocity $v$

| $\alpha=\gamma\frac{l}{v}\int_{0}^{l} \vert\vec{B}_{1}\left( z \right)\vert dz$ | (S3) |
| --- | --- |

Further assuming that $\vec{B}_{1}$ has a constant amplitude along the z-axis and is rotationally symmetric around the center of the vessel simplifies Equation (S2) to

| $\overline{M_{z}}=\frac{2}{r_{2}^{2}-r_{1}^{2}}\int_{r_{1}}^{r_{2}} r\cos\left( \frac{{\gamma B}_{1}\left( r \right)l}{v} \right)dr$ | (S4) |
| --- | --- |

When a catheter coil is centered inside a vessel the field strength decreases with distance from the center, therefore, $\overline{M_{z}}$ was solved for $B_{1}\left( r \right)=B_{1,c}\cdot1/r$ and ${B_{1}\left( r \right)=B}_{1,c}\cdot1/r^{2}$ as well as for a constant field ${B_{1}\left( r \right)=B}_{1,c}$ (solution in Appendix). The labeling efficiency $E$ was then calculated from $\overline{M_{z}}$ using Equation (2).

**Results:**

The solution of equation (S4) for the field distribution $B_{1}\left( r \right)=\overline{B}_{1}$ is:

| $\overline{M}_{z}=\frac{2}{r_{2}^{2}-r_{1}^{2}}\cos\left( \frac{{\gamma B}_{1}\left( r \right)l}{v} \right)$ | (S5) |
| --- | --- |

For $B_{1}\left( r \right)=\overline{B}_{1}\cdot1/r$ the solution is:

| $\overline{M}_{z}=\frac{2}{r_{2}^{2}-r_{1}^{2}}\frac{cos\left( \frac{a}{r_{2}} \right)r_{2}^{2}-asin \left( \frac{a}{r_{2}} \right)r_{2}+a^{2}Ci\left( \frac{a}{r_{2}} \right)}{2}-\frac{cos\left( \frac{a}{r_{1}} \right)r_{1}^{2}-asin \left( \frac{a}{r_{1}} \right)r_{1}+a^{2}Ci\left( \frac{a}{r_{1}} \right)}{2}$ | (S6) |
| --- | --- |

Finally, for $B_{1}\left( r \right)=\overline{B}_{1}\cdot1/r^{2}$, the solution is given as

| $\overline{M}_{z}=\frac{2}{r_{2}^{2}-r_{1}^{2}}\frac{\cos\left( \frac{a}{r_{2}^{2}} \right)r_{2}^{2}+a Si\left( \frac{a}{r_{2}^{2}} \right)}{2}-\frac{\cos\left( \frac{a}{r_{1}^{2}} \right)r_{1}^{2}+a Si\left( \frac{a}{r_{1}^{2}} \right)}{2}$ | (S7) |
| --- | --- |

where $a=\gamma B_{1c}t$, $Ci\left( x \right)=-\int_{x}^{\infty} \frac{\cos\left( t \right)}{t}dt$ and $Si\left( x \right)=\int_{0}^{x} \frac{\sin\left( t \right)}{t}dt$.

Figure (S1) shows $E$ calculated for rotationally symmetric fields and, for comparison, simulation results for the same fields. The results for the homogeneous $B_{1}$-field show that $E$ oscillates between 0 and 1, whereas both the radially decreasing $B_{1}$-fields show an oscillation of $E$ that decreases for larger $B_{1}$, trending towards a 0.5. Analytical calculations and the simulation show a very good agreement with $R^{2}$ > 0.91.


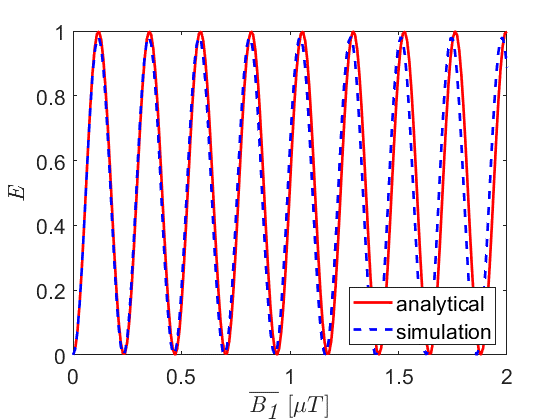

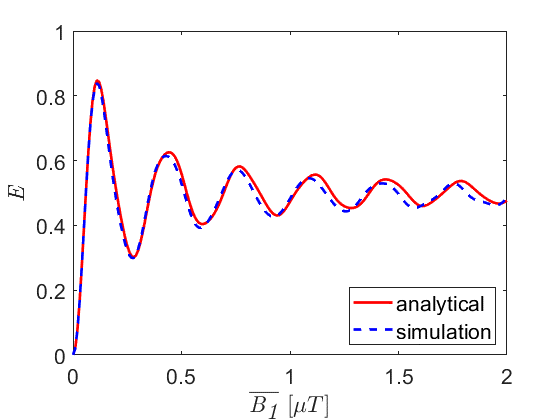

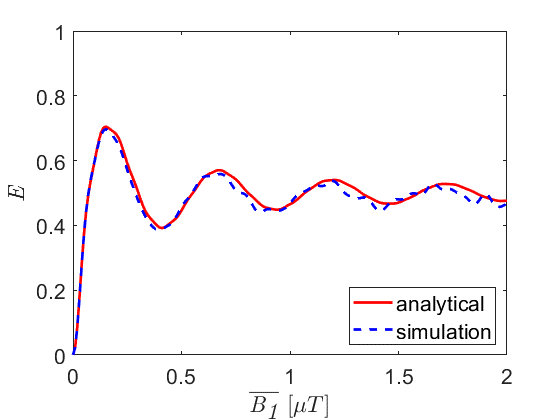

$$\boldsymbol{B}_{\boldsymbol{1}}\left( \boldsymbol{r} \right)\boldsymbol{=}\boldsymbol{B}_{\boldsymbol{1}\boldsymbol{c}}\boldsymbol{\cdot}\frac{\boldsymbol{1}}{\boldsymbol{r}}$$

$$\boldsymbol{B}_{\boldsymbol{1}}\boldsymbol{=const}$$

$$\boldsymbol{B}_{\boldsymbol{1}}\left( \boldsymbol{r} \right)\boldsymbol{=}\boldsymbol{B}_{\boldsymbol{1}\boldsymbol{c}}\boldsymbol{\cdot}\frac{\boldsymbol{1}}{\boldsymbol{r}^{\boldsymbol{2}}}$$

Figure S1 Analytically calculated labeling efficiency as function of the mean field strength in the labeling region for three different field distributions (radially symmetric around the vessel center and constant in amplitude and orientation over the length l in z direction). For comparison the simulation results for the same field distributions are shown.
